# On the (Non-)Rationality of Human Enhancement and Transhumanism

**DOI:** 10.1007/s11948-022-00410-4

**Published:** 2022-11-01

**Authors:** David M. Lyreskog, Alex McKeown

**Affiliations:** 1 NEUROSEC, Department of Psychiatry, University of Oxford, Oxford, OX3 7JX UK; 2 Wellcome Centre for Ethics and Humanities, Oxford, UK; 3 Oxford Uehiro Centre for Practical Ethics, Oxford, UK

**Keywords:** Human enhancement, Transhumanism, Transformative Experience, Rationality

## Abstract

The human enhancement debate has over the last few decades been concerned with ethical issues in methods for improving the physical, cognitive, or emotive states of individual people, and of the human species as a whole. Arguments in favour of enhancement defend it as a paradigm of rationality, presenting it as a clear-eyed, logical defence of what we stand to gain from transcending the typical limits of our species. If these arguments are correct, it appears that adults should in principle be able to make rational and informed decisions about enhancing themselves. In this paper, however, we suggest that a rational and informed choice to enhance oneself may in some cases be impossible. Drawing on L. A. Paul’s work on ‘transformative experience’, we argue that some enhancements—such as certain moral or cognitive modifications—may give rise to unbridgeable epistemic gaps in key domains. Importantly, such gaps could prove to be not merely contingently unbridgeable due to a lack of information at a given moment, but *radically* unbridgeable, making someone in a non-enhanced state inherently unable to conceive of what it would be like to be enhanced in a particular way. Where this experience is key to understanding what values are being pursued by the enhancement itself, it may prove impossible for a person to be sufficiently informed, and to make a rational decision about whether or not to enhance herself. This poses a challenge for human enhancement proponents in general, and for transhumanists in particular.

## Introduction

In this paper, our argument is that it may be in principle impossible for an agent to make the rational choice to enhance herself in certain ways. This argument rests on three premises. The first premise is that (P1) any choice is only normatively rational if sufficient substantial information is being considered by the choosing agent. The second premise is that (P2) transformative experiences by definition are such that sufficient substantial information cannot be accessed before making a decision leading to having that experience. The third premise is that (P3) human enhancements can constitute and/or give rise to transformative experiences. From these premises (P1-3) it follows that (C) it can in some cases be in principle impossible to make the rational decision to enhance oneself. In what follows, we will explicate on the three premises, argue why we believe they are true, and that (C) therefore follows.

Is should be emphasized that this enquiry primarily concerns rationality as based on personal values and preferences—what Paul calls ‘normative rational choice’—which is a quite specific form of rationality.^1^ There may be other forms of rationality at play in any given choice situation, for instance objective rationality (where X is rational, regardless of your preferences) or social rationality (X is rational because it’s what’s good for society as a whole). One could argue that these forms of rationality is what most transhumanists and human enhancement (HE) proponents are concerned with, and therefore the risk of there being a lack of rationality in terms of (personal) normative rational choice is missing the mark, as the latter is but a question of what constitutes a (subjectively) good choice *for the person*.

This paper indeed primarily addresses this specific form of rationality, as we take it to be a strong challenge to transhumanist and pro-HE positions: if certain radical enhancements cannot be said to be normatively rational in this way, then that counts against the overall rational basis for promoting or indeed pursuing such enhancements. Additionally, social and existential considerations would typically be part of the broader choice situation. While one may still claim that a certain set of enhancements (e.g., radical cognitive enhancement (CE) or moral enhancement (ME)) are important for society, or indeed for the survival of the (trans) human species, regardless of whether they appear as normatively rational for the person whom is to be enhanced, the lack of such personally normative grounds calls into question the basis for such a claim.

## Background

### Human Enhancement (HE) and Transhumanism

The core idea of human enhancement is the prospect of improving or human abilities, performance, or wellbeing by biomedical or biotechnical means (Savulescu & Bostrom, 2009; Bostrom & Roache, 2008). Typically, this is seen as an enterprise standing in contrast to that of using similar means to maintain or restore human ability, performance, or wellbeing to levels considered normal in some way—although some argue that this dichotomy could be problematic **(**Cwik, 2019; Earp et al., 2014).

Somewhat crudely, the debates around human enhancement can be decided into three categories: (1) *physical* enhancement, discussing modifications to improve performance of the human body in one way or another, through doping, bionic implants, prosthetics, and so forth (Holm & McNamee, 2011; Savulescu & Bostrom, 2009); (2) *cognitive* enhancement, looking at enhancements to our ability to focus, think, or otherwise take in and process information (Nagel, 2019; Zohny, 2015; Bostrom & Sandberg, 2009); (3) *emotive, moral,* and *motivational* enhancement, probing the implications of modifying or improving social aspects to our mental life and behaviour (Kjærsgard, 2015; Lyreskog & Nagel, 2015; Persson & Savulescu, 2012). These three areas of human enhancement have been thoroughly dissected, and intricate positions have emerged therefrom. It is not uncommon for proponents of one type of enhancement to reject another type, and to have well-grounded reasons for doing so (Chan & Harris, 2008; Harris, 2011).

It is notable that arguments in favour of any type of human enhancement typically rely on the rationality of permitting or pursuing that specific (type of) enhancement. In terms of physical enhancements (PE), life extension and anti-aging treatments to improve human longevity has been argued to be a natural and rational step to take as “this represents a wonderful opportunity to experience, learn, and achieve many things that are simply not possible given current human life expectancy” (Bostrom & Roache, 2008). Perhaps more strikingly, cognitive enhancement (CE) and moral enhancement (ME) are both commonly framed as rational necessities for the sake of the future of humanity. Technology and globalisation, it is argued, has led us to a point where it is easy to do massive harm, while we are cognitively too stupid and morally primitive to have that power (Bostrom & Sandberg, 2009; Douglas, 2008; Savulescu, 2005; Persson & Savulescu, 2012).

The idea of human enhancement as a largely rational enterprise reaches its core in the stream of ideas commonly referred to as “Transhumanism”. Proponents of this school of thought—transhumanists—distinguish themselves by adhering to a normative imperative of pursuing human enhancement to a point where humanity takes control over its evolution, and in some sense transcends into something else: a posthuman existence. Transhumanists typically argue in favour of radical forms of all three types of enhancement (1–3), and do so on the basis of it being the rational and/or natural path forward for humanity as a whole.

Overall, then, human enhancement—be it for specific settings and applications or on a broader, transhumanist scale—is generally taken to be a largely rational project, where we are assumed to be able to make informed, rational decisions about if and how to enhance ourselves. But it is far from clear that this assumption is correct.

### Transformative Experience (TE)

The phenomenon of transformative experiences has been observed and discussed in literature for quite some time but gained significant traction through the work of LA Paul in the field of decision theory, mainly publicised in her book on the subject, appropriately titled “Transformative Experience” (2014). In a nutshell, the term “transformative experience” denotes experiences which in some substantial way alter who we are and how we understand the world. Paul argues that, particularly when it comes to making significant life decisions, we may be unable to access information which is crucial to making those decisions—precisely because we lack previous experiences of what it’s like to have made those very decisions:

“The idea is that, when you find yourself facing a decision involving a new experience that is unlike any other experience you’ve had before, you can find yourself in a special sort of epistemic situation. In this sort of situation, you know very little about your possible future, [and] so, if you want to make the decision by thinking about what your lived experience would be like if you decided to undergo the experience, you have a problem. In such a situation, you find yourself facing a decision where you lack the information you need to make the decision the way you naturally want to make it—by assessing what the different possibilities would be like and choosing between them.” (Paul, 2014, p. 3).

Furthermore, Paul (2014) distinguishes between two ways in which experiences can be transformative. First, an experience can be *epistemically* transformative, in that we may learn something through the experience which would otherwise not be able to know, and consequently alter our perspective and ability to assess events and states of the world. The famous thought experiment about “What Mary didn’t know” as authored by Lewis (1988) describes such a scenario: by experiencing the colour red, Mary learns something about the world, which allows her to imagine and understand novel concepts and relationships. The experience, in this way, transforms her epistemically, as she learns something which fundamentally changes her perspective on the world. Experiences like this, it should be emphasized, are not merely epistemically *uncertain*, but epistemically *inaccessible*.^2^

Second, an experience can be *personally* transformative, in such a way that it significantly alters what it’s like to be yourself. That is, “it can change your point of view, and by extension, your personal preferences, and perhaps even change the kind of person that you are or at least take yourself to be. If an experience changes you enough to substantially change your point of view, thus substantially revising your core preferences or revising how you experience being yourself, it is a personally transformative experience.” (Paul, 2014, p. 16).

What Paul focuses on in her research, and what is especially interesting also for us here, are experiences which are *both* epistemically and personally transformative: through the experience, we learn something new about the world which transforms our point of view, and, additionally, the same experience changes our value system and how we view ourselves. However, the distinction between personally and epistemically transformative experiences does help outline what makes rational choice problematic when our choices lead to transformative experiences.

The key issue is that, on the one hand, the golden standard for rational decision-making is that decision-makers ought to choose the action that has the highest expected value; when you “want to make a rational choice between relevant alternatives, then, you must grasp, at least approximately and implicitly, the facts about the way to act that best fits your preferences.” (Paul, 2014, p. 22). On the other hand, some experiences—e.g. transformative ones—are such that you cannot access sufficient significant information to determine how the decision to undergo that very experience would end up fitting your preferences. So how can we rationally decide to go through them?

Since the publishing of Paul’s book, this problem has rippled across a number of domains, including the bioethical literature. Topics include the decision to (try to) become a parent (Paul, 2014, 2015b), understanding disability (Barnes, 2016; Paul, 2015a), and the weight of advance directives in dementia (Lyreskog et al., 2020; Walsh, 2020). So far, however, this debate has not spread to the literature on the ethics and normative frameworks of human enhancement and transhumanism. This is surprising, as some enhancements—particularly, but not exclusively, those which align with the transhumanist agenda—arguably will lead to substantial transformative experiences.

With this in mind, we here argue that it under some circumstances may be in principle impossible for a person to make the rational decision to undergo enhancements. The argument rests on three premises:

- (P1) Any choice is only normatively rational if sufficient substantial information is being considered by the choosing agent;
- (P2) Transformative experiences by definition are such that sufficient substantial information cannot be accessed before making a decision leading to having that experience;
- (P3) Human enhancements can constitute and/or give rise to transformative experiences.

In what follows, we shall outline more explicitly what these three premises entail, and why we hold them to be true.

## Choosing to Enhance

### Rational Normative Decision-Making

Premise 1: Normative choice is only rational if sufficient substantial information is being considered by the choosing agent.

This is the first premise of our argument, and while strictly a matter of definition, it could do with some justification and clarification. In essence, Premise 1 (P1) implies that it could only be that I *should* choose something if I have sufficient substantial reasons to expect that this choice will lead to a given value being successfully pursued. This makes the issue a subject of *normative* decision theory, to be contrasted with *descriptive* decision theory, where the latter seeks to describe how decisions are (de facto) made, rather than how they ought to be made. A sound normative decision theory is important as “when we make decisions, we want to make them rationally, at least as rationally as we can, and normative decision theory gives us the models and the principles for the procedures we should follow—as some describe it, it is “action-guiding.”(Paul, 2014) Adopted by most normative decision theorists, is the idea that agents ought to choose the act which has the highest expected value. That is, when deliberating whether to choose one action or another, the normatively rational thing to do is to compare the probabilities and expected outcomes of those actions, and subsequently choose the one which has the highest expected value.

The key thing here, for our investigation, is the accessibility of sufficient substantial information about the probability and nature of the outcomes of those actions to be able reasonably meet some sort of standard for normative, rational, choice. Is it the case that we under epistemically challenging circumstance may not be able to make a normatively rational choice? Let us look at an example.

Imagine that you are invited to participate in a gameshow where you can make a lot of money. Your goal with participating is to make as much money as possible during your appearance. In the show you are asked by the host to select one of two opaque boxes. You are told that they both contain money, but one contains significantly more than the other. Both boxes have the same volume, but one box is orange, fluffy and makes a vague noise, and the other box is purple, shiny, and makes a loud noise. Which one should you choose?

You have been given information about the boxes, and about how they differ from one another, and yet the question seems silly. It cannot be that you *should* choose the orange box over the purple box, or vice versa, the reason being that you only have useless information, given your goal of making as much money as possible. In other words, you do not have sufficient substantial information to make a normatively rational choice. If more information emerges, however, the situation might change. Perhaps the host informs you that the fluffier the box, the more likely it is to contain a high price. Now you have some substantial information. But is it sufficient? There may be further information to gain. For instance, you may be informed that purple boxes on the show always contain the higher price. Now, instead, the normatively rational choice is for you to pick the purple box, as you have sufficient substantial information about what to expect, and what your given goal is.

In this way, normatively rational decision-making—i.e., what we *should* do in a given choice scenario—depends on our access to sufficient substantial information about our goal, and about the probability of possible choices leading to that goal. If we cannot know enough about (a) what the outcome of the choice is (epistemic information), or (b) what our personal disposition towards that outcome is (personal information), we cannot rationally choose it for the sake of achieving that outcome. In this way, it is reasonable to hold that Premise 1 (P1) is correct: Normative choice is only rational if sufficient substantial information is being considered by the choosing agent.

### What Mary Cannot Rationally Choose

Premise 2 of our argument is: (P2) Transformative experiences are by definition such that sufficient substantial information cannot be accessed before making a decision leading to having that experience.

This, again, is strictly speaking a matter of definition, but it does raise the question: can we—and, indeed, *do* we—make decisions where the consequences are in this way inaccessible to us? The question is whether it can be rational to choose something so transformational that, by definition, one cannot make the decision based on a reliable anticipation of what it will be like and how it will affect or alter one in undergoing it. This challenge can be interpreted in at least two ways, however. One interpretation is that choosing to have a transformative experience is no less rational than any other choice the first time it is made, given that everything is radically unknowable before that point. For example, one could not know what it is like to learn to swim until one has attempted it. That it could not be known does not necessarily make the choice irrational, however, partly because, on the basis of what we know about it, the experience of something like swimming does not appear to be transformational. Of course, there will be people who discover, on learning to swim, that they have a passion for it in such a way that it directs further life choices and transforms their life in this respect. It is not, *in general*, however, an experience which usually has such a profound impact that the person who has just swum for the first time cannot identify with themselves prior to having done so.

Moreover, the activity of swimming is, as we have indicated above, sufficiently commonplace and easy to observe, so that although the first-person experience is unknowable before having done it, enough information can be gathered about what the outcome of going swimming is likely to be for one to make an informed choice to do so. In this sense, therefore, the unknowability of going swimming does not undermine the possibility of deciding rationally that one ought to go for a swim, if doing so is likely to be satisfying, based on the comprehensive information that one can collect about it and the relatively low risk of harm or other deleterious outcomes. We will develop this point further in the next section.

The other interpretation is that *some* experiences are *so* transformational that it is in the nature of the experience that it precludes the possibility of acquiring substantial information in advance about what one’s experience of the world will be like after one has experienced it. This is to say, there is a category of events characterised by their having an impact so qualitatively dramatic that these changes would have been beyond the horizon of imagination before an individual decides to experience them. Unlike something like swimming, a transformative experience is one with such a profound impact that once having had it, one would recognise the testimony or observation of others who had it as falling far short of communicating what the experience is like.

There may, therefore, be some kinds of events which are never *fully* rational to choose, in the sense that one could never know enough about the experience to go ahead with it in a comprehensively informed way. Indeed, the transformational potential of these events are in part characterised by their being far beyond the boundary of imagination.

### On Parenthood, Dementia, and Space Travel

What kind of experiences are like this? Paul argues that many—but not all—of our big choices in life may lead to transformative experiences which are problematic from a rational decision point of view. One such choice is to pursue parenthood:

“[W]hether the primary basis for your new phenomenology is simply the experience of producing a child, or the particularized experience of producing this particular child, or your first experience of parental love, or being in new physical states that realize new conscious states—or whether it is some complex mix that includes all of these—when you become a parent, you have an experience you’ve never had before, an experience with an epistemically unique character, and in the normal case, one that generates a sustained, intense, felt attachment to the child.

Now, having a child is not just a radically new experience. For many people, it is also a life-changing experience. It might be wonderful, or joyous, or happy—or it might not. However, it is usually very intense, and people who have a child and respond in the normal way find themselves with very different perspectives and preferences after the child is born. That is, for most people, having a child is an epistemically transformative experience that is also personally transformative. Your preferences will change. The way you live your life will change. What and who you care about will change.” (Paul, 2014, pp. 80–81).

In this way, Paul argues, having a child is a transformative choice, changing how we view the world and who we are. The experience of having a child would therefore be so transformative that normatively rational choice—choosing something based on the value we expect from making that choice—becomes problematic.

This problem appears also in other domains of life. For instance, it becomes painfully clear in the context of dementia and advance directives (Lyreskog et al., 2020; Walsh, 2020). Walsh (2020) convincingly argues that the process of progressive dementia is epistemically and personally transformative, as it alters our cognition and in extension our perspective on life as well as our views on ourselves and others. The implication is that advance directives—legal documents which state our wishes about what should happen to us once we are deemed “incompetent” with regard to medical decision-making—should have little to no bearing: how can we ethically make decisions for our future self, living with dementia, when that future self is epistemically and personally inaccessible to us? (Walsh, 2020).

Another well-documented transformative experience, known as the ‘Overview Effect’, has been reported by astronauts and is instructive for thinking through transformation in the context of human enhancement. Despite still being relatively rare, and certainly much less common than becoming a parent or developing dementia, the transformative impact of going to space is not the entirely unknowable experience that it was prior to the 1960s. In this regard it may be helpful to think about it as analogous to viewing certain kinds of cognitive or moral enhancement retrospectively from the vantage point of the future, looking back to the point at which humans first developed the means to have those experiences. The experience of seeing the Earth from space, whether or not from the vantage point of the Moon, frequently (Weibel, 2020) gives rise to a phenomenon which, although described in different ways, is characterised by transcendence of the usual human viewpoint and a way of conceiving “almost all that is meaningful in human life; it has tremendous, perhaps absolute, conceptual vastness. Seeing it from a distance, when one is disconnected physically yet connected emotionally, conjures thoughts of home, of the entirety of one’s world, and of mankind as a whole” (Yaden et al., 2016).

Testimonies of astronauts themselves give the most effective account of what this phenomenon is like. Crucially, the experience of seeing Earth, on which until the advent of space travel all human history had taken place, has been described as providing unique moral insights into the self and its relation to the rest of humankind and nature. Key to the inscrutability of the overview effect, it is characterised by resulting from *“*an atypical method of engaging with the natural environment…it is one that offers demonstrable and perhaps enhanced benefits in terms of promoting self-transcendence, eudaimonic well-being and feelings of connectedness with nature”.

Drawing on White’s (1998, 2021) extensive research interviewing astronauts about the impact that seeing the Earth from space had on them, Bjornvig (2013, p. 5) summarises their experiences as *“*…the impact of the view of Earth seen from space on both astronauts and humankind at large. Ultimately, it triggers a new stage in human evolution. It can transform a person’s reality and entails the realization that Earth is a star-orbiting spaceship traveling through the galaxy. It is connected to changed ways of perceiving space and time along with intense experiences of silence and weightlessness”.

Clearly, different individuals do not necessarily have identical reactions to experiences and instances of the Overview Effect reflect this. For example, in relation to the moral dimension of the transformation that occurs, it has caused some individuals to suffer. Buzz Aldrin (2010) found that although his experience was profound in its taking him beyond the horizon of what he could previously imagine, after being one of the first humans on the Moon, he found that *"there was no goal, no sense of calling, no project worth pouring myself into"*, and struggled with alcoholism for many years.

Even though very few people are astronauts, since the Overview Effect is well-documented, uncertainty about what seeing the Earth from space can be reduced. However, the transformative nature of the experience does not appear to be diminished for astronauts by there having been prior astronauts reporting this set of phenomena. Indeed, in interviewing astronauts about their experiences of the overview effect, Nezami (2017, p. 196) found that even though the research involved necessarily a small subset of all previous astronauts, there were commonalities in the profound influence that the effect had on them. Across the cohort, it was found that “earth gained value beyond just its form, usefulness, and affordances; and transformed into multiple attachment objects, a mother, a life force, and a sacred object worthy of respect and honour, love and compassion. This strengthened their loyalty and impetus to protect Earth. It seems in order to grasp and integrate the uncertainty, the participants perceived the universe as purposive and having a numinous quality suggesting some may believe in a universal spirit, or perhaps a higher consciousness or power”.

The reduction in uncertainty does not undermine the epistemic unavailability of the experience, and furthermore, in bringing about the kind of profound perspectival shifts that it reportedly does, there is a sense in which one could never know enough about of what to expect, as one’s understanding of what the shift entails alters by that very same shift.

Considering these cases above, it appears as if some choices we make in life are such that sufficient substantial information about what it will be like—epistemically and personally—cannot be accessed before making the decision. By extension, this poses a problem from a normative, rational point of view: arguably, we cannot rationally choose to undergo an experience X based on our value set V, if that set V is insufficient to assess the impact of X, and the only way of appropriately updating our value set is to undergo X.^3^,^4^

### Transformative Experiences and Human Enhancement

The third and final premise of our argument is: (P3) human enhancements can constitute and/or give rise to transformative experiences.

Given the preceding analysis, there are reasons to think that certain kinds of human enhancement modifications would qualify as *both* epistemically *and* personally transformative. This is crucial, as the predictability of the effects of radical enhancements, should they become possible, will be an important determinant of access and availability. This is to say, there are straightforward reasons to exercise more caution in the case of very radical enhancements than for more modest ones. Enhancements of the kind envisaged in the former, transformational, category that we will focus on would include dramatic increases in cognitive capacity or moral sensibility.

Certainly, extreme physical modifications may also be transformational since, for example, Paul (2014) identifies losing a limb as a physical change that may transform one’s experience in ways that could not be known beforehand. However, there is significant qualitative data available about what it is like to be physically altered in ways such as this (Coffey et al., 2009; Liu et al., 2010; Senra et al., 2012), whereas hitherto there has been no human with, for example, an IQ of 500.^5^ As such, we are in a similar epistemic position to humans prior to the first astronauts going into space—there is no data whatsoever that we can use even heuristically in deciding what conditions should obtain in allowing access to extreme cognitive or moral enhancements.

Moreover, if Martens (2019) is correct, humans are in general not very good at predicting what will satisfy their preferences; as such, this line of argument may further undermine the possibility that one could rationally choose something truly beyond the horizon of comprehensibility. Taking a sceptical line but from a different direction, implicit in Agar’s (2014) view is that transformative enhancement would be *ir*rational because the further one has experiences beyond the normal range, the less meaningful for us they will be because we would be commensurately less able to engage with them. He concludes that these experiences would be *‘less prudentially valuable than the experiences that they would replace’*. Indeed, Agar (2014) regards radical enhancement as a negative transformational change, even if the transformed state was to be qualitatively ‘good’ in some respects: if the change constitutes a definitive break with norms of human existence, he argues, it cannot logically be held to be something that it is rational for a human *qua* a human to choose. It may be good for the transformed being, but this is not the same as being good in terms of being a human; and if the experience compromises the transformed person’s ability to comprehend life in normal human society, then to choose it would be to lose something worthwhile rather than to gain it. As such, if his argument holds, it is reasonable to infer that it would not be rational to choose such experiences, since it would not be rational to choose something less valuable than what it is one already has.

The problem with this position is that since values differ, what is regarded as an objective standard of value by Agar or anyone else may be less valuable, in a pre-enhanced state at least, than stepping into the truly unknown, whatever the consequences, for someone else. Nevertheless, divergence does not undermine the premise that certain human enhancements might give rise to transformative experiences; and moreover, it does not follow from the fact that some people may *prioritise* experiencing the truly unknown that it is *rational* to do so.

### Rationally Choosing Enhancement

Leaving aside for the moment whether it is rational, in the prudential sense, to choose radical enhancement, it is not especially difficult to make the case that such enhancements are probably transformative. For example, in his discussion of machine intelligence and posthuman ethics, Bostrom (2014) has invited us to try to conceive the difference in experience between humans and beetles and then to try to conceive the difference between an enhanced human that bears the same relation in cognitive sophistication to us as we do to those beetles. In neither case can the experience be imagined. Developing the theme of posthuman and/or machine intelligence, or hybrids of the two (Benedikter & Fathi, 2019), much has been written in the relevant literature about mind uploading (Blackford & Broderick, 2014; Kurzweil, 2005;). Instances such as these, where one no longer has a body in a conventional sense, in which one is to all intents and purposes immortal (Huberman, 2018), and in which one has the opportunity of become continuous with other minds, becoming capable of collective cognition by removing the barrier of the human body that encase the brain, are impossible to comprehend (McKeown, 2021). While this may sound like science fiction, advances in development of brain-computer interfaces and brain-brain interfaces are rapidly moving us closer to a point where we will need clear frameworks for what counts as merely a collection of individual minds, and what is to be regarded as a collective mind—a hivemind (Jiang et al., 2019; Maksimenko et al., 2019; Zhang et al., 2019). As such, any enhancement of the kinds outlined here is highly likely to be transformational. Indeed, Paul (2014) refers to the experiential differences between humans and other animals in a similar way. It is beyond the horizon of predictability to grasp what it would be like to be cognisant in the way that another animal is; as such, to think in this way would be transformative.

Similarly, the argument has been made in the moral enhancement literature that to prevent the demise of the human species we should undergo moral enhancement that to allow us to have the same degree and kind of empathy for humans far beyond our own in-group as we do for those close to us, so that we act in the interests of all of humanity, rather than a limited circle of humans who we know and who we are likely to prioritise (Persson & Savulescu, 2012). The idea here is that the capacity for empathy is crucial for moral capacity, and that widening the scope for that empathy by extension would allow an improved moral capacity. Indeed, an even stronger position has been advanced that it is unimportant whether ‘we’ remain recognisably human, if moral enhancement helps to prevent the end of civilization (Persson & Savulescu, 2010). Given that imagining what this kind of moral sensibility would be like is beyond the horizon of our current capacities, as with radical cognitive enhancement, this kind of modification is also highly likely to be transformative. In support of this conclusion are reports from persons with reduced moral capacities, e.g. persons with anti-social personality disorder, or “psychopaths”.

One person, who was diagnosed with psychopathy, explained in an interview with The Cut: “People think we have no emotion, which is absolutely not true. We just feel them way turned down. If most people feel an emotion between seven and eight on a dial of ten, I feel it between zero and two. Negative emotions are background noise. We can’t tune into that frequency because our brains just don’t process enough information for them to ever be loud enough to feel or direct behaviour.”(The Cut, 2018).

Kent Kiehl, expert on psychopathy and violent recidivism, was once asked what it was like meeting with patients scoring 40 out of 40 on the Hare psychopathy scale,^6^ and responded: “They are so fundamentally different. You leave the room knowing that you've just met someone who is extremely different, even different from other psychopaths. They are absolutely and completely free from conscience. They have this unbelievably flat affect that's really palpable when you look in their eyes.” (Wired, 2014).

In this way, we can see that moral capacity is a matter of degree, which one can in principle possess to a higher or lesser degree. While the interviewee in the first case may or may not undergo a transformative experience were s/he to gain moral capacity at a baseline level (whatever we take that to be), the psychopath Kiehl describes as “extremely different” is arguably likely to do so: a completely new dimension of being and experiencing the world would emerge, amounting to both empirical transformation (in gaining the ability to process moral information) and personal transformation (in changing who they are as a person).

If we can imagine an equivalent shift in moral capacity from a “normal” to an “enhanced” state of being, to the radical level suggested by Persson and Savulescu (2010, 2012), it is quite plausible that also this would lead to a transformative experience. If so, it seems that it cannot be normatively rational to choose to enhance oneself in that way.

It seems, then, that:

1. Any choice is only normatively rational if sufficient substantial information is being considered by the choosing agent;
2. Transformative experiences by definition are such that sufficient substantial information cannot be accessed by an agent before making a decision leading to having that experience;
3. Human enhancements can constitute and/or give rise to transformative experiences.

If these three premises hold, as we have argued, it follows that:

(C) *It can in some cases be in principle impossible for an agent to make the normatively rational decision to enhance oneself*.

Any objection to this conclusion will need to deny one or several premises (1–3) or show why they do not lead to (C). But to the authors of this paper, it seems as if the premises are true, following the arguments laid out above, and that (C) indeed follows from them.

## Towards Transformative Enhancements

It should again be emphasized that we do not argue that all or even most enhancements will give rise to transformative experiences. What we do argue is that some forms of enhancement—such as substantial moral enhancement or the formation of hive minds—may give rise to such experiences. Furthermore, we do not argue that enhancements leading to transformative experiences are inherently irrational in all respects. We are strictly limiting our argument to that of normative rational choosing as defined above, entailing the key mechanism of pursuing maximum value. Usually this would make rational and informed choice impossible given that the most obvious reasons for enhancing are frustrated by epistemic inaccessibility. However, there may be other (good or bad) reasons to choose to go through with these enhancements, which are part of a wider range of rationality. For instance, it may be rational to enhance oneself in these ways if the main aim with doing so is to explore certain mental states, to change oneself for the sole purpose of changing, or to conform to a certain social group whose members have also enhanced themselves in these ways. However, these other reasons seem largely absent in the literature promoting human enhancement and transhumanism. Therefore, significant enhancements giving rise to transformative experiences remains a concern for transhumanists and other proponents of radical forms of human enhancement.^7^

That being said, it is still unclear what the full normative implications of this position are, beyond the theoretical discomfort it may cause radical enhancement proponents and transhumanists. What should we do about transformative enhancements on a practical level? One may be inclined to argue that, given that normative rational choice is likely to be unachievable in cases of significant moral enhancement or hivemind-like enhancements, informed consent is impossible, and therefore such enhancements should be prohibited across the board. The authors of this paper doubt that this is an adequate policy response. In parallel cases of transformative choice, some of which we have outlined above, including parenthood and moon journeys, it is not our intuition that these choices should be prohibited. By extension, it is therefore unclear why transformative enhancements should be prohibited for this reason—we are free to make other transformative choices, so why not the decision to enhance ourselves? Unless other substantial harms are to be expected there seems to be few reasons why prohibition of transformative enhancements is an appropriate response.

However, arguably some form of standardized and improved decision-making processes should be established to ensure that persons aiming to enhance themselves substantially are supported by a larger network of co-decision-makers, increasing the likelihood of positive outcomes—whichever they may be. In many countries we already have forums for persons who have made or are about to make transformative choices, where more elaborate decision-making processes are enabled and facilitated. For instance, groups for family planning and parenthood are widely established (McLeish & Redshaw, 2017; Solomon et al., 2001**)**, and shared supported decision-making systems for dementia sufferers and caregivers are being developed (Daly et al., 2018; Span et al., 2014) In this way, we can learn from other areas of society and in the health care sector to establish appropriate frameworks and improve our position ahead of substantial human enhancements.

## Conclusion

In this paper, we have argued: (C) It can in some cases be in principle impossible for an agent to make the normatively rational decision to enhance oneself. We have sought to show how this follows from three premises: (P1) Rational choice is only possible with sufficient substantial information being considered by the choosing agent; (P2) Transformative experiences are by definition such that sufficient substantial information cannot be accessed before making a decision leading to having that experience, and (P3) Human enhancements can constitute and/or give rise to transformative experiences.

We have argued for why we believe that all three premises hold, and that the conclusion (C) indeed follows. Any objection to this argument will need to show that at least one of the premises (P1-P3) is incorrect, or show why they do not lead to (C). We have additionally suggested that, while this alone may not warrant a prohibition of transformative enhancements, it poses a problem for transhumanist and enhancement proponents who mainly argue from the point rationality. Furthermore, we argued that, as human enhancements continue to become increasingly sophisticated and potent, it is vital that we seriously consider the informed consent processes leading up to such enhancements, and that we think hard about how to deal with epistemic gaps, such as those brought about transformative experiences.

## Footnotes

1 More on this in our paragraph regarding Premise 1 below.

2 Paul elaborates on the importance of this specification in her response to Pettigrew’s critique [Pettigrew, 2015; Paul, 2015a, b].

3 Presumably, the exceptions to this would be cases where discovery or experience of transformative events is at the core of the value set. One may argue that this is plausible in the case of psychedelic or spiritual experiences, for instance, where stepping beyond the epistemic horizon is a key reason for having the experience in the first place. Such reasons, however, are largely absent in the literature on human enhancement and transhumanism. In the case of transformative experiences in the use of psychedelics, we can look at Earp’s (2018) analysis of the role of such in moral enhancement. He draws a distinction between their having a determinative and an adjunctive role in moral enhancement. The former refers to using psychedelics without preparation for what will happen, and they *cause* moral enhancement; the latter refers to using them after preparation and learning from experts about what is likely to happen, such that they *facilitate* moral enhancement. Leaving aside uncertainty about how meaningful a distinction this is to draw, both cases are consistent with our argument, since, assuming use is *deliberate and chosen*, rather than accidental, the openness to experiencing something which one knows will be, at least, highly unusual, indicates that a desire for mind-altering experiences is part of a person’s core value set. In this respect, there is scope to argue that going beyond the threshold can be a rational choice even if, in the other sense of rationality at play here, there is no way to know what the experience will be like.

4 One may argue this goes both ways, regardless of what choice we make: in choosing *not* to undergo X, we are also in the dark, in that we lack sufficient substantial information about what the option (to undergo X) would entail. The main difference here seems to be in the rationality of remaining in a familiar value system: I can rationally choose to not-X, because I am familiar with what not-X entails and what my values are with relation to not-X. I.e., there is an expected outcome which I can rationally pursue because I have enough lived experience of it. There is perhaps an argument to be made that one cannot rationally choose not-X if one only knows what not-X entails, and does not know what any other available option entails. This, however, would need to be shown.

5 We acknowledge that IQ is a somewhat crude measure of ‘intelligence’, since it tests only a relatively narrow,—albeit important—range of capacities, and indicates no more than where an individual lies in a normal distribution for those capacities. We use IQ as an example only to point out that the experience of someone with an IQ of 500 would be inscrutable to the vast majority of other people, and it is in this sense that it is illustrative for our argument, in that transformative experiences exist beyond a threshold which cannot be properly comprehended without having gone over it.

6 The Hare psychopathy scale is a widely used assessment tool for identifying psychopathic traits in people (Hare, 1991).

7 Transhumanists or proponents of radical human enhancement may of course respond with a ‘so what’ objection here: that we cannot rationally choose something does not mean that the choice will have bad consequences, for instance. While this may be true, this stance is largely absent in the literature and—as argued in the introduction to this paper—proponents typically implicitly or explicitly appeal to the rationality of the endeavour. For any project which leans on rationality as a key argument in favour of it, we contend that the impossibility of rationally choosing to participate in or contribute to it poses a problem.
